# Altered white matter integrity in individuals with cognitive vulnerability to depression: a tract-based spatial statistics study

**DOI:** 10.1038/srep09738

**Published:** 2015-05-18

**Authors:** Jing Xiao, Yini He, Chad M. McWhinnie, Shuqiao Yao

**Affiliations:** 1 Beijing Key Laboratory of Learning and Cognition and Department of Psychology, Capital Normal University, Beijing 100048, China; 2 Harvard Medical School, Boston, MA, USA; 3 Medical Psychological Research Center, Second Xiangya Hospital, Central South University, Changsha, 410011, China; 4 Hunan Province Technology, Institute of Psychiatry, Changsha, Hunan, 410011, China

## Abstract

The microstructure of white matter in patients with major depressive disorder (MDD) has been demonstrated to be abnormal. However, it remains unclear whether these changes exist prior to the onset of disease. In this study, diffusion tensor imaging was used to evaluate white matter integrity in individuals who exhibited cognitive vulnerability to depression (CVD), MDD, and healthy controls (HC). Compared with the HC, MDD exhibited a lower fractional anisotropy (FA) in ten brain regions: the cerebral peduncle, the anterior and posterior limbs of the internal capsule (ALIC and PLIC), the external capsule, the retrolenticular part of the internal capsule (RLIC), the body and splenium of the corpus callosum, the superior and posterior corona radiata, and the cingulum. Moreover, CVD had significantly lower FA in the ALIC, the PLIC, the external capsule, the RLIC, the cerebral peduncle, and the superior corona radiata than did the HC. However, the white matter integrity was not significantly different between the CVD and MDD. These preliminary results indicate that alterations in the white matter observed in CVD may be a marker of vulnerability to MDD and that these alterations may exist prior to the onset of depression.

In recent years, researchers have attempted to better understand why some individuals but not others become depressed following stressful life events. In particular, a model of cognitive vulnerability to depression (CVD) has been developed^1^, in which relatively stable aspects of an individual predispose him or her to develop depression following negative life events^2^. In this model, depression is the result of interactions between cognitive vulnerability factors (e.g., diatheses) and certain environmental conditions (e.g., stressors)^2^. The results of the Temple-Wisconsin CVD project have suggested that in the high-cognitive vulnerability group, the lifetime prevalence rate of major depressive disorder (MDD) is 38.7%. The risk of lifetime MDD in the high-cognitive vulnerability group was threefold that of the low-cognitive vulnerability group^3^.

One of the most prominent CVD models is the hopelessness theory^4^. This theory defines cognitive vulnerability as the tendency of an individual to make particular types of inference regarding causes and consequences. Specifically, when faced with a negative life event, an individual who has a cognitive vulnerability is likely to (1) attribute negative events to stable, global causes; (2) to perceive negative events as having many disastrous consequences; and (3) to infer negative characteristics about the self after negative events^5^. These characteristics further increase the likelihood that hopelessness will develop, which can lead to the onset or recurrence of depression^6^,^7^,^8^. A growing number of cross-sectional and prospective studies support the theory of CVD^9^,^10^,^11^.

Recently, various neuroimaging and histological techniques have been used to examine neural mechanisms in the pathogenesis of depression. In these studies, depressed individuals exhibited structural and functional neural abnormalities compared with non-depressed individuals. Moreover, cortical-subcortical neural circuits were found to underlie the neurobiology of depression, especially the frontal-striatal-thalamic and limbic-thalamic-frontal networks^12^,^13^,^14^,^15^. Limited structural and functional imaging data from individuals with cognitive vulnerability also support these models^16^,^17^,^18^. More specifically^17^, functional magnetic resonance imaging scans were performed while participants with MDD or CVD and healthy control (HC) participants engaged in an emotional face matching paradigm. In response to negative emotional faces, the MDD group exhibited increased left amygdala responses and decreased left dorsolateral prefrontal cortex (DLPFC) responses relative to the HC group. These findings may explain the abnormalities identified in the neural networks that mediate the cognitive modulation of emotions in individuals with CVD. In a study by Nusslock et al. (2011), increased CVD was associated with decreased relative left frontal brain activity at rest in individuals with no prior history or current experience with depression using electroencephalograms. Zhang and colleagues (2012) also compared MDD, CVD, and HC groups using voxel-based morphometry to assess structural differences in the brain. Significant volumetric differences were identified among the three groups in the left precentral gyrus, the right fusiform gyrus, and the right thalamus. It was hypothesised that these findings correlated with negative cognitive styles, as well as an increased risk of depression.

Diffusion tensor imaging (DTI) is a non-invasive method used to investigate the orientation and integration of white matter microstructure *in vivo* via measurements of the diffusion characteristics of water in neural tissue. A commonly used metric in DTI is fractional anisotropy (FA)^19^. FA reflects aspects of the membrane integrity and myelin thickness, with decreased FA associated with the disruption of white matter^15^,^20^. Decreased FA has been identified in samples obtained from individuals with depression^21^, as well as subjects with genetic or familial vulnerabilities to depression^15^,^22^,^23^. To date, a direct comparison of DTI changes between individuals with MDD and CVD has not been performed. Moreover, it is not clear whether the white matter of individuals with CVD undergoes changes that precede the occurrence or development of depression. This issue is an important area for future research, particularly because white matter abnormalities may represent vulnerability markers for MDD, which could thus identify individuals at greater risk for depression. Therefore, in this study, DTI was used to evaluate and compare the white matter integrity in individuals who exhibited CVD or MDD and in healthy controls.

## Results

**Figure 1:**
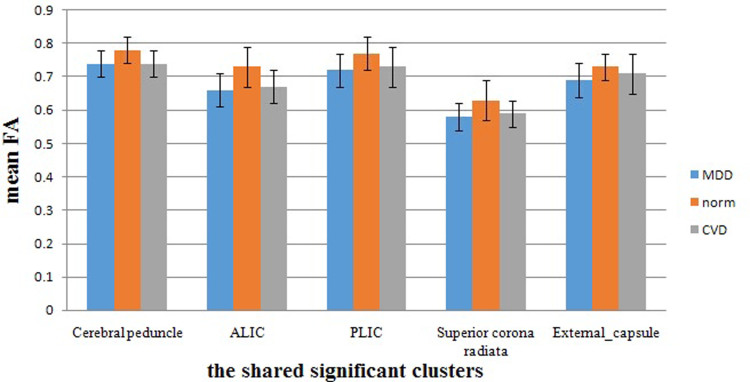
The mean FA values across the MDD, CVD and healthy control groups for shared significant clusters in the TBSS.

**Figure 2:**
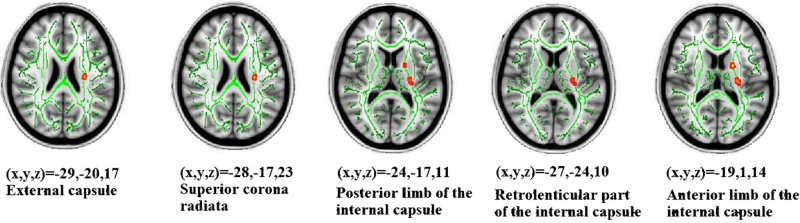
An ANOVA was used to compare the individuals with CVD, those with MDD and the healthy controls, and the significant clusters are shown (*p* < 0.01, voxel > 50). The background image is a standard MNI152 1 × 1 × 1 mm^3^ brain template. Green voxels represent the mean FA identified for the white matter skeleton, and the red-yellow voxels represent the regions in which the FA significantly differed between the MDD, CVD and healthy control groups.

**Figure 3:**
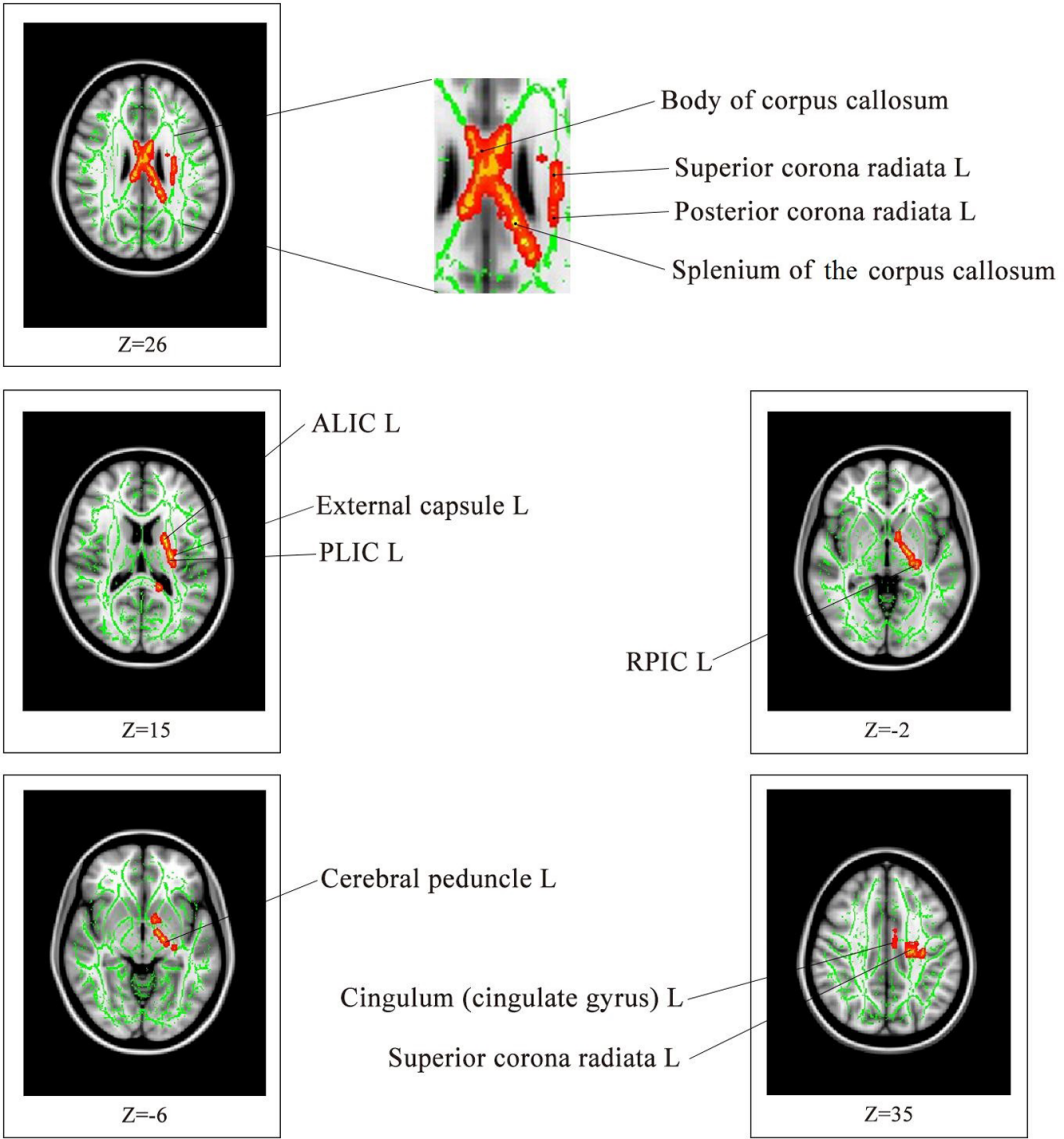
Compared with the healthy controls, the individuals with MDD exhibited significantly lower FA values in ten brain regions (*p* < 0.001, corrected for multiple comparisons). The background image is a standard MNI152 1 × 1 × 1 mm^3^ brain template. The green voxels represent the mean FA detected for the white matter skeleton, and the red-yellow voxels represent regions where the FA is significantly lower in the individuals with MDD than in the healthy controls. ALIC: anterior limb of the internal capsule; PLIC: posterior limb of the internal capsule; RLIC: retrolenticular part of the internal capsule.

**Figure 4:**
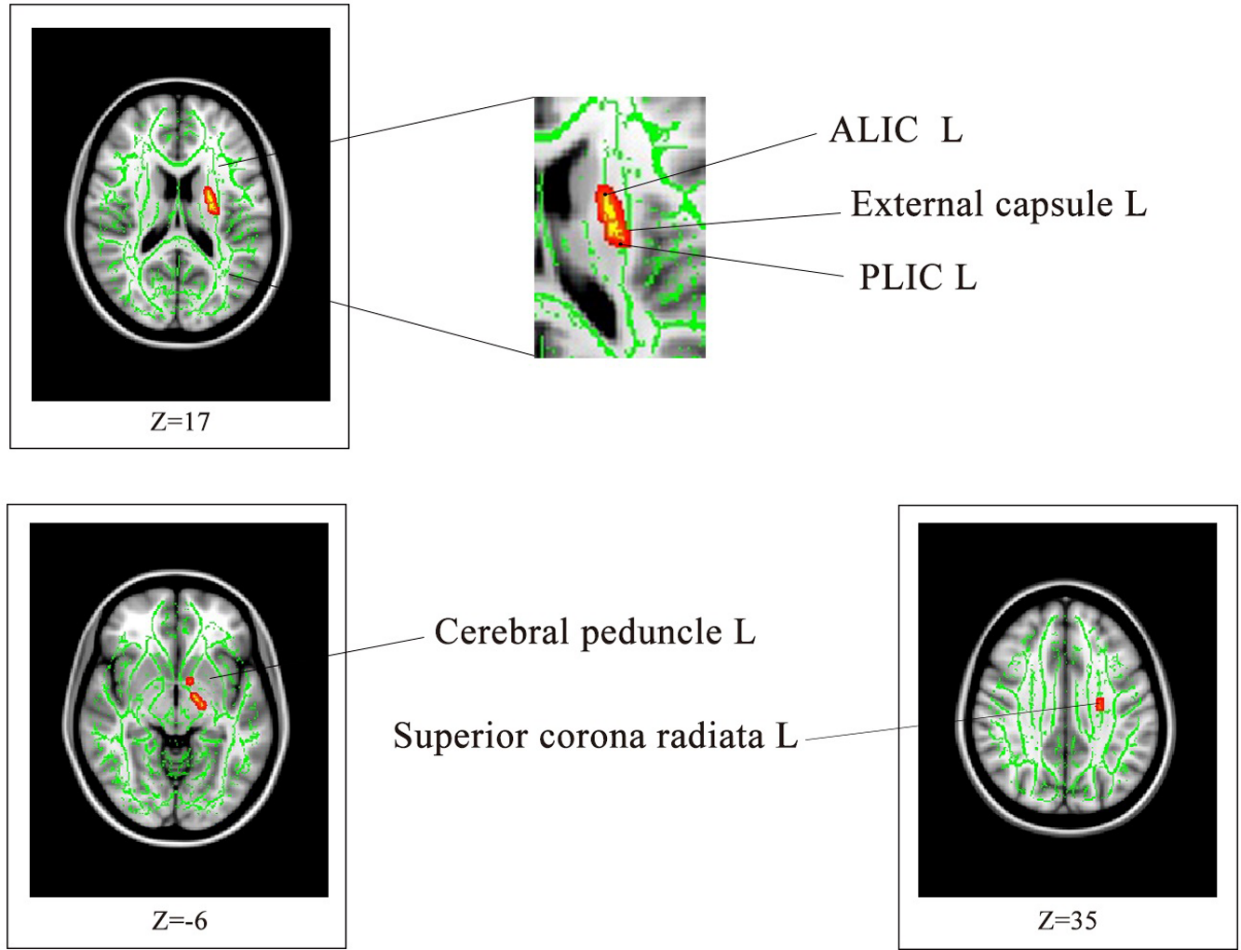
Compared with the healthy controls, the individuals with CVD exhibited significantly lower FA values in six brain regions *(p < 0.05*, corrected for multiple comparisons). The background image is a standard MNI152 1 × 1 × 1 mm^3^ brain template. The green voxels represent the mean FA detected for the white matter skeleton, and the red-yellow voxels represent regions where the FA is significantly lower in the individuals with CVD than in the healthy controls. ALIC: anterior limb of the internal capsule; PLIC: posterior limb of the internal capsule.

The demographic characteristics of the participants in this study are listed in Table 1. The effect size/mean FA + SD value of the common significant clusters in the individuals with MDD/CVD compared with the healthy controls are shown in Table 2 and Figure 1. The three groups were not significantly different in terms of age, race, gender, or years of education. An ANOVA was used to compare the results of the CVD, MDD and healthy control groups, and the significant clusters are shown in Figure 2 (p < 0.01, voxel > 50). Specifically, a significantly lower FA was identified in the individuals with MDD than in the healthy controls (Table 3, Figure 3). Aberrant FA values were identified in ten regions of the brain: the cerebral peduncle, the anterior limb of the internal capsule (ALIC), the external capsule, the posterior limb of the internal capsule (PLIC), the retrolenticular part of the internal capsule (RLIC), the body and splenium of the corpus callosum, the superior and posterior corona radiata, and the cingulum (*p* < 0.001, corrected for multiple testing). Conversely, a significantly lower FA was identified for the individuals with CVD than for the controls (Table 4, Figure 4). The regions involved in the latter effect included the following: the cerebral peduncle, the ALIC, the external capsule, the PLIC, the RLIC, and the superior corona radiata (*p* < 0.05, corrected for multiple testing. Both the comparison between the individuals with MDD and the healthy controls and between the individuals with CVD and the healthy controls identified these five significantly different clusters, Figure 1). Finally, none of the white matter tracts was associated with the sociodemographic or clinical variables, and there were no differences in the white matter FA values between the CVD and MDD groups. The FA of the in the left ALIC tended to correlate negatively with the Center for Epidemiologic Studies Depression Scale (CES-D) scores (*r* = −0.43, *p* < 0.05) in the MDD group. No other correlation was identified between the FA values of the significant regions and the CES-D scores. We also found no correlation between the FA values of the significant regions and the weakest link scores.

## Discussion

To the best of our knowledge, this is the first study to report white matter tract differences in individuals with CVD. Specifically, the current findings indicate that individuals with MDD and CVD both manifest changes in the ALIC, PLIC, RPIC, cerebral peduncle, and superior corona radiata compared with HC individuals. Moreover, these changes were present before the occurrence and development of depression.

The ALIC has received attention in depression research owing to the structural and functional abnormalities that have been reported for these connected regions to ALIC in depression^24^,^25^,^26^. In the present study, compared with the HC individuals, the individuals with MDD or CVD exhibited a significantly lower FA in the ALIC, which is consistent with accumulating evidence suggesting that damage to the frontal-subcortical circuits underlies the pathophysiology of MDD^12^,^27^,^28^. Moreover, neuroimaging studies show that the medial forebrain bundle (MFB) demonstrates a clear interaction with the reward system, which mediates feelings and expectations of pleasure^29^. Moreover, the supero-lateral medial forebrain bundle (slMFB) extends from the main trunk of the ventral tegmental area (VTA). This branch shears out laterally, travels ventral to the thalamus and ascends to the inferior portion of the ALIC. The slMFB may be involved in the neurobiology of depression^25^ and is becoming an increasingly important region for deep brain stimulation (DBS) in treatment-resistant MDD. Furthermore, given that white matter abnormalities have been shown to contribute to a disconnection syndrome between the frontal and subcortical regions and may represent a risk factor for affective disorders^21^, decreased activity in these regions may also impair the ability of individuals to block out negative thoughts^30^.

The cortical and subcortical areas in the frontal lobe, anterior limb, and genu of the internal capsule are included in the prefrontal subcortical circuit, whereas the cortical subcortical areas in the PLIC are part of the fronto-limbic-striatal circuit^16^. These two circuits play important roles in the formation of emotions, cognition and behaviour control^9^, and their roles in primary depression and post-stroke depression have also been investigated. In the present study, both the individuals with CVD and those with MDD exhibited a significantly lower FA in the PLIC, and this finding may account for the cognitive style of individuals with CVD.

In a study of subjects with mild cognitive impairment (MCI) by Zhuang^31^, the left superior corona radiata exhibited lower FA values, thereby suggesting that white matter changes in this brain region may serve as a potential biomarker of MCI. In the present study, the individuals with CVD or MDD also exhibited a significantly higher FA in the superior corona radiata. Thus, this white matter region may contribute to one of the neural mechanisms responsible for CVD.

The external capsule provides a route for cholinergic fibres from the basal forebrain to the frontal, temporal, and parietal cortical areas^32^. Therefore, it is highly relevant for executive functions, cognitive control, and emotion regulation^33^. The lower FA values identified in the external capsule in the present study may indicate a disruption of white matter integrity. These results are consistent with previous studies in which lower FA values in the external capsule were identified in MDD patients than in healthy controls^25^,^34^. Taken together, these results suggest that an abnormal decrease in the FA of the external capsule may be related to affect cognitive functions and increase vulnerability to depression.

In conclusion, the results from the current study support the hypothesis that alterations in the white matter microstructure observed in individuals with CVD may be a marker of vulnerability to MDD, and these alterations may exist prior to the onset of disease. Longitudinal studies with larger sample sizes are needed in future studies to confirm the observed microstructural white matter changes and to provide additional mechanistic details about the relationship between alterations in the white matter microstructure and CVD. It is anticipated that these insights will aid in the development of effective prevention and treatment programs for depression.

## Methods

### Individuals with cognitive vulnerability to depression (CVD)

Individuals were screened using the weakest link approach to determine a cognitive vulnerability score (CVS) for each participant. This approach posits that an individual's most depressogenic vulnerability is the best marker of his/her true propensity to develop depression^34^. To compute the weakest link composite score for each participant, the participants' scores were standardised based on three inferential styles [standardised score = (data score - mean)/standard deviation (SD)]^34^. The highest of the three standardised scores was designated as the individual's weakest link score^35^. The study participants comprised a subset of individuals selected to participate in our CVD Project. The final dataset included 595 undergraduates with a mean weakest link score of 0.42 (SD = 0.93). The CVD group included the participants with scores greater than 1.35 (one SD above the mean of the full group, 0.42 + 0.93), resulting in the identification of 22 individuals with CVD (10 males, 12 females). The selected individuals were subsequently interviewed by two experienced psychiatrists using the Structured Clinical Interview for the DSM-IV (SCID)^36^ to verify that they were free of affective disorders and other current Axis I disorders. None of the enrolled individuals had a history of major medical or psychiatric illness.

### Individuals with MDD

A total of 22 undergraduates with MDD (10 males, 12 females) who had recently experienced their first depressive episode were referred for therapy and recruited as outpatients from the psychology clinic at the Second Xiangya Hospital of Central South University. A diagnosis of MDD was confirmed by two trained psychiatrists using the SCID. None of these patients had previously been diagnosed with an Axis I disorder or received psychiatric medication prior to DTI.

### Healthy controls (HC)

Similar to the CVD individuals, the HC individuals were also selected based on their Cognitive Style Questionnaire (CSQ) results. The HC group included 22 gender and age-matched individuals (10 males, 12 females) who had received weakest link scores of less than 1.35. These individuals were subsequently interviewed by two experienced psychiatrists to verify they were free of affective disorders and other current Axis I disorders. None of the individuals had a history of major medical or psychiatric illness.

To ensure homogeneity across the three groups, all individuals were 18–24 years of age and had a similar education status. Conversely, individuals were excluded if they had the following medical conditions: significant self-reported medical conditions that could have a significant impact on cognitive function, a lifetime history of substance dependence, substance abuse within the last six months, or self-reported central nervous system disorders (e.g., head injury, seizure disorder, or multiple sclerosis). In addition, all enrolled individuals had normal or corrected-to-normal vision and were right-handed. This study was conducted in accordance with the Declaration of Helsinki and was approved by by the Ethics Committee of the Second Xiangya Hospital of Central South University (No: CSMC-201118). All individuals provided written informed consent prior to participation in the study.

### Measures

Cognitive Style Questionnaire (CSQ). Abramson and Metalsky created the CSQ based on the hopelessness theory to assess the depressogenic cognitive style. It contains 24 items associated with causes, consequences, and the self. The score for each item ranges from 1 to 7, and increased scores correspond to a more depressogenic cognitive style. In the present study, the Cronbach's alpha for the CSQ subscales ranged from 0.90 to 0.91, suggesting strong internal consistency. Previous studies have also documented the ability of the CSQ to assess the depressogenic cognitive style in Western countries^3^,^34^ and mainland China^18^,^37^.

Center for Epidemiological Studies Depression Scale (CES-D). This scale was developed by Radloff^38^ and contains 20 items designed to assess depressive symptoms in the general population. The total raw scores can range from 20–80, and increased scores indicate greater levels of depressive symptoms. The Chinese version of the CES-D has been demonstrated to exhibit a high degree of reliability and validity^39^. In the present study, a Cronbach's alpha value of 0.91 was obtained for the total scale, indicating strong internal consistency.

Edinburgh Handedness Inventory (EHI). The EHI was developed by Oldfield^40^ and is a questionnaire designed to evaluate the handedness of the participants.

Structured Clinical Interview for the DSM-IV (SCID). The SCID is a semi-structured interview that includes modules designed to assess an individual's history and current (within the past month) experience of categorically defined DSM-IV Axis I psychiatric disorders (including mood and anxiety disorders). Currently, the SCID is the “gold standard” for psychiatric diagnosis of MDD. For Chinese patients with mood disorders, the interrater reliability for the SCID diagnoses has a kappa value of 0.76^41^. All participants were interviewed prior to DTI by two psychiatrists who have experience with the SCID.

### Image acquisition and processing

All individuals underwent a DTI exam at Central South University (Changsha, Hunan, China) using a 1.5-T Siemens Magnetom Symphony scanner and standard head coil. The individuals were provided ear plugs and foam pads to reduce noise and decrease head motion, respectively. The diffusion tensor data were received using a coronal diffusion-weighted, single-shot, spin-echo planar (SE-EPI) imaging sequence parallel to the line of the anterior-posterior commissural. The parameters for this imaging method included: scanned field of view (FOV) = 24 cm, matrix of the exported image = 128 * 128, repetition time (TR) = 12,000 ms, echo time (ET) = 105 ms, excitation frequency (EF) = 5, slice thickness = 4 mm, no gap, and 30 adjoining axial slices. Diffusion-sensitising gradients were applied along 13 non-combining directions (b = 1000 s/mm^2^) and without diffusion weighting (b = 0 s/mm^2^). DCM2NII software (http://www.cabiatl.com/mricro/mricron/dcm2nii.html) was used to convert the raw DICOM files from the proprietary scanner format to the nifti format, “.image”. The diffusion-weighted images were analysed using the Functional Magnetic Resonance Imaging of the Brain Library (FSL, Oxford, United Kingdom). Briefly, the FSL eddy-correct tool was used to register all diffuse images in the B0 image space. The FSL bet2 was then used to skull-strip the brain to ensure that only tensors inside the brain were calculated, rather than those in the surrounding air, using a threshold of 0.25. Finally, a FSL DTIFIT tool was applied to calculate the diffusion tensor model at each pixel and obtain a FA image map. After data processing, tract-based spatial statistics (TBSS)^42^ were used to explore the group differences between the white matter skeletons derived from the FA images. Nonlinear registration was used to align all FA images to a standard space template. A mean FA skeleton, which represented the centres of all tracts common to the group, was then generated. Each individual's aligned FA image was projected onto the mean FA skeleton, and an FA threshold of 0.20 was established to exclude the peripheral tracts. Finally, group differences in FA among the MDD, CVD and healthy controls were assessed using an ANOVA implemented in the FSL randomize function (5000 permutations) of the TBSS. Then, correction for multiple comparisons was conducted using a cluster-based thresholding method with an initial cluster that formed a threshold at clusters with at least 50 voxels and *p* < 0.01 or *p* < 0.05. To label the significant clusters from the TBSS analysis, we used the digital white matter atlas JHU ICBM-DTI-81, which identified 48 white matter tract labels by hand segmentation of a standard-space average of the diffusion MRI tensor maps from 81 normal subjects (see http://fsl.fmrib.ox.ac.uk/fsl/fslwiki/Atlases). As shown in Figures 2, 3, and 4, the JHU WM atlas was overlaid on the WM skeleton of each subject in the ICBM-DTI-81 space, and the white matter clusters were subsequently labelled.

## Author Contributions

S.Q.Y. and X.J. designed the research. X.J. and H.Y.N. performed the experiment, collected the data and analysed the data. Chad M.M contributed to the analytic tools. H.Y.N. and X.J. prepared the figures and wrote the manuscript.

## Additional Information

**How to cite this article**: Xiao, J., He, Y., McWhinnie, C.M. & Yao, S. Altered white matter integrity in individuals with cognitive vulnerability to depression: a tract-based spatial statistics study. *Sci. Rep.* 5, 9738; DOI:10.1038/srep09738 (2015).

## Figures and Tables

**Table 1:** Demographic parameters and scores for the analysed cohort

Demographic parameters	HC (n = 22)	MDD (n = 22)	CVD (n = 22)
Age (y)	20.77 ± 1.41	20.14 ± 1.64	20.59 ± 0.91
Gender (M/F)	12/10	12/10	12/10
Education (y)	13.62 + 1.41	13.84 ± 1.10	13.59 ± 0.80
CESD scores	39.72 ± 7.75	55.68 ± 5.75	40.55 ± 9.60
Weakest link scores	1.07 ± 0.40	1.10 ± 0.61	2.28 ± 0.37

HC, healthy controls; MDD, major depressive disorder; CVD, cognitive vulnerability to depression; CESD, Center for Epidemiologic Studies Depression Scale.

**Table 2:** The effect sizes in the individuals with MDD/CVD compared with the healthy controls

Regions of the brain analysed	X*	Y*	Z*	Cohen'd
**MDD vs. healthy control**				
Cerebral peduncle L	−16	−15	−6	−1.00
Anterior limb of the internal capsule L	−20	−2	15	−1.26
External capsule L	−29	−13	15	−1.00
Posterior limb of the internal capsule L	−26	−18	15	−0.98
Superior corona radiata L	−28	−12	26	−0.66
**CVD vs. healthy control**				
Cerebral peduncle L	−16	−15	−6	−1.00
Anterior limb of the internal capsule L	−20	−2	15	−1.09
External capsule L	−29	−13	15	−0.72
Posterior limb of the internal capsule L	−26	−18	15	−0.78
Superior corona radiata L	−28	−12	26	−0.39

*: X, Y, and Z stereotaxic coordinates are given in the Montreal Neurological Institute (MNI) space.

**Table 3:** FA values in the individuals with MDD compared with the healthy controls

Regions of the brain analysed	X*	Y*	Z*	*p*-value	Voxel size
Cerebral peduncle	−16	−15	−6	0.001	1534
Anterior limb of the internal capsule	−20	−2	15	0.001	
External capsule L	−29	−13	15	0.001	
Posterior limb of the internal capsule L	−26	−18	15	0.001	
Retrolenticular part of the internal capsule L	−24	−22	−2	0.001	
Body of corpus callosum	3	−2	26	0.001	928
Splenium of the corpus callosum	−14	−33	26	0.001	
Superior corona radiata L	−28	−12	26	0.001	
Posterior corona radiata L	−27	−26	26	0.001	
Cingulum	−7	−8	38	0.001	

*: X, Y, and Z stereotaxic coordinates are given in the Montreal Neurological Institute (MNI) space.

**Table 4:** FA values in the individuals with CVD compared with the healthy controls

Regions of the brain analysed	X*	Y*	Z*	*p-*value	Voxel size
Anterior limb of the internal capsule L	−22	−3	17	0.05	1543
External capsule L	−25	−10	17	0.05	
Posterior limb of the internal capsule L	−26	−18	15	0.05	
Cerebral peduncle L	−27	−29	17	0.05	
Superior corona radiata L	−14	−13	−6	0.05	

*: X, Y, and Z stereotaxic coordinates are given in the Montreal Neurological Institute (MNI) space.
